# Separation and Determination of Theobromine and Caffeine in Cocoa Beans Extract Using TLC‐SERS: Identification and Computational Insights

**DOI:** 10.1002/ansa.70033

**Published:** 2025-07-28

**Authors:** Maria Rodriguez, Ray Arteaga, Briggit Katan, Maria Figueira, Romel Guzman, Castillo Jimmy

**Affiliations:** ^1^ Escuela De Quimica Universidad Central de Venezuela Los Chaguaramos, Caracas DC Venezuela; ^2^ Instituto De Ciencia y Tecnología De Alimentos Escuela De Biologia Universidad Central de Venezuela Los Chaguaramos, Caracas DC Venezuela

## Abstract

In recent years there has been a growing interest in cocoa and their sub products in the world, given the beneficial properties of these products. This interest has led to increased research in the study of the composition of cocoa and its relationship with its varieties, mainly the principal alkaloids, theobromine and caffeine. Venezuela, although a small‐scale producer, is recognised worldwide for the quality of its cocoa. This work presents a robust, unambiguous and cost‐effective methodology for the rapid and accurate quantification of theobromine and caffeine in cocoa beans extracts from Venezuelan cocoa. Thin layer chromatography (TLC) is used for separation, and alkaloids are identified by their Rf values and by their Raman spectra obtained by surface‐enhanced Raman spectroscopy (SERS). In the SERS technique, the spots of separated compounds by TLC were impregnated with a solution of silver nanoparticles and the SERS spectra record. Given the great structural similarity of these alkaloids, principal component analysis (PCA) was used to show that despite the similarities of the Raman spectra, they are perfectly distinguishable. Theoretical calculations were performed using Orca software, obtaining Raman and FTIR spectra, and similarities were found between the theoretical and experimental responses, validating the computational approach. The synergistic integration of TLC for separation, SERS for sensitive detection, PCA for robust differentiation and DFT for theoretical validation offers a cost‐effective, rapid and robust analytical platform for the unambiguous identification of theobromine and caffeine in complex matrices. This methodology lays the foundation for future quantitative applications in the evaluation of cocoa quality and origin.

## 1 Introduction

Interest in cocoa and its derivatives has increased considerably in recent years, driven by its use in the food industry as a confectionery and nutritional supplement, as well as its recognised health benefits [1]. This trend has generated growing scientific interest in the chemical and physicochemical characterisation of these products. Venezuela, despite being a small‐scale cacao producer and exporter, enjoys worldwide recognition for the exceptional quality of its cacao. Varieties such as Criollo, Forastero and Trinitario are highly valued for their unique organoleptic characteristics, which derive directly from their complex chemical composition [1, 2, 3]. In addition to the compounds that give it its organoleptic properties, alkaloids such as theobromine and caffeine are of great interest due to their antioxidant properties and other health benefits. The chemical composition and in special ratio of these bioactive compounds is particularly significant for the classification and commercialisation of cocoa, driving the need for rapid, accurate and cost‐effective methodology of analysis of these compounds [4, 5, 6, 7].

Chemical analysis of cocoa and its derivatives include the determination of organic and inorganic components. In the case of inorganic, it mainly is focusing on metals considered micronutrients as iron, calcium, magnesium and so on, and heavy metals such as cadmium and mercury, which are considered as contaminants, are typically analysed by techniques such as optical inductively couple plasma (ICP), with mass detector (ICP‐MS) and graphite furnace or flame spectroscopy [8, 9, 10, 11, 12, 13]. The organic components including the separation and determination of a complex mixture of compounds, is analysed by methods like gas chromatography (GC), GC‐mass spectrometry (GC‐MS) and high‐performance liquid chromatography (HPLC) [1, 2, 3, 7, 14, 15, 16, 17]. Amongst the organic components that give it these unique organoleptic properties, alkaloids such as theobromine and caffeine are of particular interest due to their important properties [18, 19].

Whilst these techniques are reliable, they are often expensive, require extensive sample preparation and long analysis times, limiting their applicability in resource‐limited settings or for rapid on‐site quality control. Thin‐layer chromatography (TLC) is a well‐established technique for separation a mixture of organic compounds. The separation is based on their differential affinity for the stationary and mobile phases, resulting in the developed of distinct spots on the TLC plate [20]. Modern TLC techniques, such as high‐performance TLC (HPTLC) [16], have significantly improved its resolution and applicability [1, 2, 3, 4, 5]. One of the tools used in TLC for the identification of the different components in a sample is the comparison with standards through their retention factor (Rf), which is the ratio of the distance travelled by the sample compared to the distance travelled by the solvent front. However, definitive identification based solely on Rf values can be ambiguous, especially when compounds with very close Rf values coexist or in complex matrices. To address this limitation, TLC can be coupled with spectroscopic techniques such as infrared (IR), Raman and mass spectrometry (MS) for compound‐specific identification [16, 20, 21, 22, 23, 24]. Raman spectroscopy is a technique with great sensitivity and versatility for molecular analysis, in the case of molecules with similar characteristics such as caffeine and theobromine, where the difference at the molecular level is determined by a methyl group, Raman spectroscopy serves as a technique for differentiation without any doubt between these molecules. Previous works have studied and characterised the Raman spectra of different components in cocoa [20, 25, 26, 27], finding spectral differences between compounds such as caffeine and theobromine. This characterisation allows to differentiate without any doubt these compounds in cocoa samples, as well as to allow the development of techniques for both qualitative and quantitative analysis of the samples.

The technique, surface‐enhanced Raman spectroscopy (SERS) is a highly sensitive technique to study vibrational energies in organic molecules. The Raman signal is significantly enhanced when molecules are adsorbed or in close proximity to nanoparticles of noble metal surfaces, such as silver or gold nanoparticles. The effect is under discussion, for different authors the enhancement is primarily due to the excitation of localised surface plasmons, which amplify the electromagnetic field at the metal surface [15, 16, 17, 18]. Additional enhancement mechanisms, such as chemical and resonance effects, can further increase sensitivity, enabling detection at the single‐molecule level under ideal conditions [19]. The combination of TLC with SERS (TLC‐SERS) offers great potential for the separation and sensitive detection of analytes. TLC‐SERS has been applied to various chemical species, including amino acids, pharmaceuticals and historical artefacts [8, 10]. Although the silica substrate of TLC plates does not significantly interfere with the SERS signal, it produces an intense background signal that needs to be corrected, and the interactions between the Si─OH groups and analytes can lead to hydrogen bonding, potentially causing shifts in the observed spectra [28]. The combination of TLC with SERS (TLC‐SERS) offers great potential for the sensitive separation and detection of analytes and has been applied to a variety of chemical species, including amino acids, pharmaceuticals and so on.

Theoretical studies of the energy of molecules and their interaction with electromagnetic field have been used in understanding spectroscopic properties of organic molecules [28, 29, 30] and the comparison with experimental values. The use of computational methods based in molecular dynamics calculations with density functional theory (DFT) with Orca software allowing the prediction of molecular vibrations and consequently the IR and Raman vibrational spectra, aiding in the interpretation of experimental results. The identification of structurally similar compounds using spectroscopic techniques faces challenges due to overlapping spectral features, particularly in molecules like caffeine and theobromine, which share analogous chemical frameworks. To address this limitation, multivariate statistical methods such as principal component analysis (PCA) have emerged as powerful tools to help to analyse and differentiate complex spectral data. PCA reduces multidimensional datasets into principal components that capture the maximum variance, enabling the visualisation and differentiation of small spectral differences imperceptible through conventional analysis. This approach has been successfully applied in alkaloids or plant metabolites, by highlighting unique spectral fingerprints [31, 32]. In this work, PCA was used to validate and distinguish identity of caffeine and theobromine, despite their spectral similitude in both Raman and FTIR analyses. By leveraging PCA, the work aligns with recent advancements in chemometrics that emphasise the integration of computational and experimental strategies for accurate compound characterisation [33].

This work focuses on developing a methodology to analyse cocoa bean extracts by separating molecules from the mixture using TLC and identifying them using SERS. To ensure the reliability of the methodology, the obtained spectra were analysed using PCA, and calculations were performed using Orca software [34, 35] for comparison and analysis of the experimental results [1]. The integration of these techniques aims to develop a robust, cost‐effective and rapid methodology for the accurate identification of theobromine and caffeine in cocoa, contributing to the assessment of cocoa quality and origin [1]. The term ‘determination’ in the title of this work refers to the unequivocal identification of theobromine and caffeine through their unique spectral fingerprints, a critical initial step for subsequent quantitative analysis. The economic viability and scalability of the proposed method are key aspects that reinforce its analytical significance. The synthesis of silver nanoparticles (AgNPs) is carried out from low‐cost precursors (AgNO_3_, NaBH_4_), and TLC plates are significantly more cost‐effective compared to HPLC columns or GC–MS consumables. In terms of instrumentation, Raman spectrometers, especially portable models, are increasingly affordable and offer lower maintenance costs than HPLC/GC–MS systems. From a scalability perspective, TLC allows for simultaneous analysis of multiple samples on a single plate, reducing the time per sample. SERS detection can be automated with micropositioning stages for rapid spot scanning. Furthermore, compatibility with portable Raman systems opens the possibility of on‐site quality control at cocoa farms or production facilities, avoiding the need for laboratory‐dependent techniques. This combination of factors makes the methodology particularly relevant for cocoa quality control, especially in regions where access to high‐end instrumentation is limited.

## 2 Materials and Methods

### 2.1 Materials

The reagents used in this investigation were silver nitrate (99%), sodium borohydride (NaBH_4_) (98+%), caffeine (99%) and theobromine (98%), all of which were purchased from Sigma‐Aldrich (Dorset, UK). Carboxymethyl cellulose (99%), ethyl formate, formic acid, toluene and ethyl acetate from Aldrich.

### 2.2 Synthesis of Silver Nanoparticles

AgNPs were synthesised by reducing AgNO_3_ using an aqueous solution of NaBH_4_ as the reducing agent, following a modified version of the method reported by Mavani and Shah [36]. In brief, 25 mL of a 10^−3^ M AgNO_3_ solution was added dropwise to 75 mL of a 2 × 10^−3^ M NaBH_4_ solution, ice cooled, vigorously stirred. The reaction produced a yellow colloidal suspension of AgNPs. No stabilising or protective agents were added to avoid interference with the Raman signal. The resulting colloid was stable when stored in the dark at room temperature. To ensure maximum consistency and reproducibility of results, freshly prepared AgNP solutions were used for each experiment, thus mitigating any potential long‐term degradation effects, although these nanoparticles have been shown to be stable for periods exceeding 1 week.

### 2.3 Sample Characterisation

The synthesised AgNPs were characterised using the following techniques:

The average diameter and particle size distribution of the AgNPs were determined using a DLS system developed in the Laser Spectroscopy Laboratory at UCV. A small amount of the nanoparticle suspension was dispersed in ultrapure water and sonicated for 10 min. The dispersed sample was then analysed using the DLS equipment. AFM analysis was performed using a Bruker Dimension Edge instrument operated in tapping mode. Silicon nitride cantilevers were used with scanning speeds ranging from 0.5 to 1 Hz, depending on the scanned area. To prepare the samples, 1000 ppm carboxymethyl cellulose (CMC) solution is deposited onto a silicon slide and left dry, after that, a small amount of the nanoparticle suspension was dispersed in the silicon modified slide. The silicon slide recover with CMC was dried in an oven at 100°C to ensure adhesion of the nanoparticles to the surface. The extinction spectrum of the AgNPs dispersed in water was done using an Ocean Optics USB2000 UV‐Vis spectrophotometer. The sample was prepared by dispersing a small portion of the nanoparticle suspension in 5 mL of distilled water, followed by sonication for 5 min. FTIR spectra were acquired using a PerkinElmer Spectrum Two spectrometer by depositing a thin film of the sample onto a potassium bromide (KBr) pellet and allowing it to dry. SEM analysis was conducted to examine the morphology of the AgNPs. Samples were prepared by depositing a drop of the nanoparticle suspension onto a silicon wafer and allowing it to dry at room temperature.

### 2.4 Thin‐Layer Chromatography (TLC)

TLC was employed for the separation of caffeine and theobromine. The methodology was as follows: Stock solutions of caffeine (2–200 mg/L), in 50% aqueous acetone and theobromine (20–300 mg/L) in methanol were prepared. Aliquots (1 µL) of the standard solutions were applied to a silica gel TLC plate using a 2 µL capillary. The plate was pre‐saturated for 15 min in a chromatographic tank containing a developing solvent system. Two different system were employed: (a) ethyl formate/formic acid/water/toluene (10:1.33:1:1:0.5 v/v); (b) ethyl acetate/methanol/water (10:1.35:1 v/v) to compare separation. The plate was then developed until the solvent front reached the desired height. After development, the TLC plate was dried, and the separated spots were visualised under UV light at 254 nm. The Rf values for each compound were calculated. For SERS analysis, 4 µL of the AgNP colloid was deposited directly onto the marked spots on the TLC plate. The spots were then allowed to dry before Raman measurements. The selection of the solvent system was crucial to achieving optimal separation. Several solvent mixtures previously reported in the literature were evaluated for this application. System A (ethyl formate/formic acid/water/toluene) was chosen for its superior performance, evidenced by a great difference in Rf values between caffeine and theobromine (Table 1). This greater difference in Rf values resulted in a more sensitive separation and a significant reduction in the uncertainty in the determination of the compounds in the sample.

**TABLE 1:** Rf values for TLC elution.

Sample	Rf caffeine	Rf theobromine	Rf sample spot 1	Rf sample spot 2
A	0.84	0.71	0.85	0.70
B	0.48	0.43	0.51	0.43

### 2.5 SERS Detection

SERS measurements were performed using a Raman spectrometer (Thunder Optics, model Avaspec Mini2048CL‐SAR4) equipped with a 532 nm excitation laser. The thunder optics equipment is composed of a laser of 532 nm wavelength and a maximum power of 250 mW, the laser is guided by an optical fibre to an objective lens of 20× magnification to focus on the sample. The Raman signal emitted by the sample together with the laser scattering is received by the objective lens and passes through a filter that absorbs the scattered laser radiation and collimates the rest to an optical fibre that directs it to the Avaspec Mini 2048CL spectrometer. The spectrometer has 2400 L/mm and a resolution of 0.20 nm. The following parameters were used: 1 s per spectrum. The laser power was adjusted to maximise the signal while avoiding photodegradation of the sample. Although the system has a maximum power of 200 mW, measurements were performed at lower operating powers (e.g., 60 mW for dark coloured samples and even lower powers for colourless samples), maintaining a constant power for each series of experiments. SERS spectra were acquired directly from the TLC plate after deposition of the AgNPs. To ensure statistical robustness and reproducibility of the results, all SERS measurements were performed in triplicate and the signal averaged.

### 2.6 Calibration Protocol

Standard solutions of theobromine (20–300 mg/L) and caffeine (2–200 mg/L) were prepared and 2 µL of the solution are deposited in TLC plate, and 10 µL AgNPs is added before SERS measurement. Post‐TLC separation, spots were dissolved in 1 mL methanol, 10 µL AgNPs are added before measurement, and analysed via SERS. Peak intensity between 1490 and 1700 cm^−1^ was integrate and plotted against concentration for calibration.

### 2.7 Computational Details

Molecular dynamics calculations with DFT were performed using the ORCA program (version 6) [34, 37] at the r2SCAN‐3c level of theory. The r2SCAN‐3c method employs the re‐regularised meta‐GGA functional r2SCAN with the Def2‐mTZVPP basis set. The calculations included geometrical counterpoise (gCP) and D4 dispersion corrections to account for basis set superposition errors and dispersion interactions, respectively. The resolution of identity (RI) approximation was applied to accelerate the computations. The choice of the r2SCAN‐3c method was based on its balance between accuracy and computational efficiency, which is particularly advantageous for the prediction of vibrational spectra. This functional is known for its ability to handle dispersion interactions and basis set superposition errors more robustly than alternatives such as B3LYP or PBE0, a critical aspect for modelling weak interactions such as those occurring between the analyte and AgNPs in SERS. Furthermore, the Def2‐mTZVPP basis set provides near triple‐zeta quality at a reduced computational cost, which is critical for the feasibility of calculations in systems and the size of the molecules studied. Reference studies have shown that r2SCAN‐3c outperforms other methods in predicting IR/Raman frequencies for alkaloids, with mean absolute errors (MAE) below 10 cm^−1^, validating its suitability for this type of analysis.

## 3 Results and Discussion

### 3.1 Characterisation of Silver Nanoparticles (AgNPs)

**FIGURE 1:**
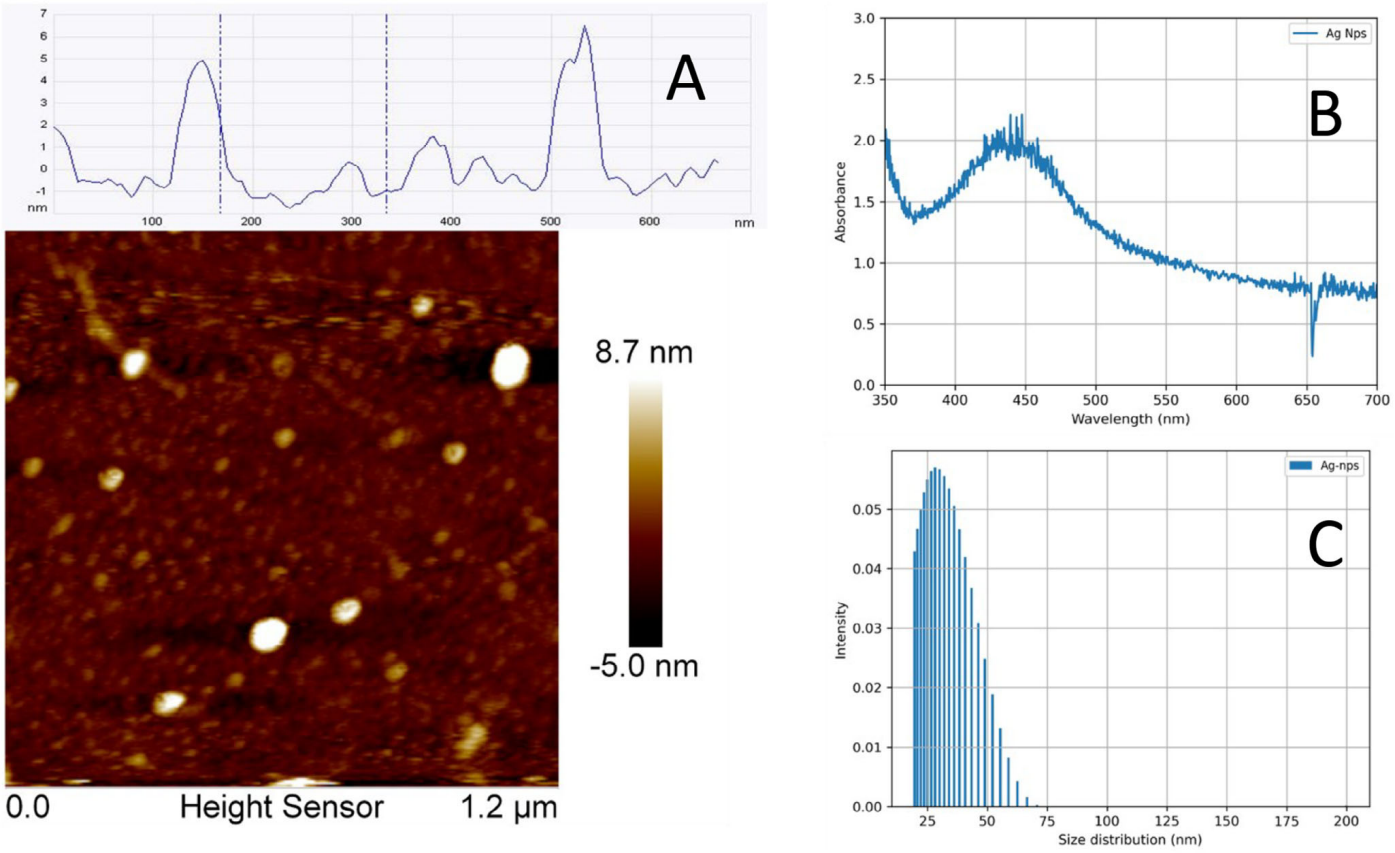
Nanoparticle characterisation, (A) AFM image of nanoparticles with high profile showing the size between 6 and 8 nm. (B) UV‐Vis spectra showing the plasmon surface at 430 nm. (C) DLS plot showing the hydrodynamic size distribution.

The AgNPs synthesised by the proposed method, were characterised using atomic force microscopy (AFM) to determinate the form and size, UV‐Vis spectroscopy and dynamic light scattering (DLS) to determine the hydrodynamic diameter of the particles. Figure 1A shows the AFM image of the nanoparticles, the intensity profile shown particles with diameters ranging between 4 and 9 nm. Figure 1B displays the UV‐Vis extinction spectrum of the AgNPs solution, showing a characteristic plasmon resonance peak at 430 nm with a full width at half maximum (FWHM) of 80 nm. This peak is indicative of spherical AgNPs with an average size of approximately 20 nm, consistent with previous reports in the literature. Figure 1C presents the particle size distribution measured by DLS, indicating an average hydrodynamic radius of 28 nm. These results confirm that the AgNPs synthesised for SERS experiments have an average size of around 20 nm, with a distribution ranging from 10 to 30 nm, making them suitable for SERS applications.

### 3.2 Separation and Detection of Theobromine and Caffeine

**FIGURE 2:**
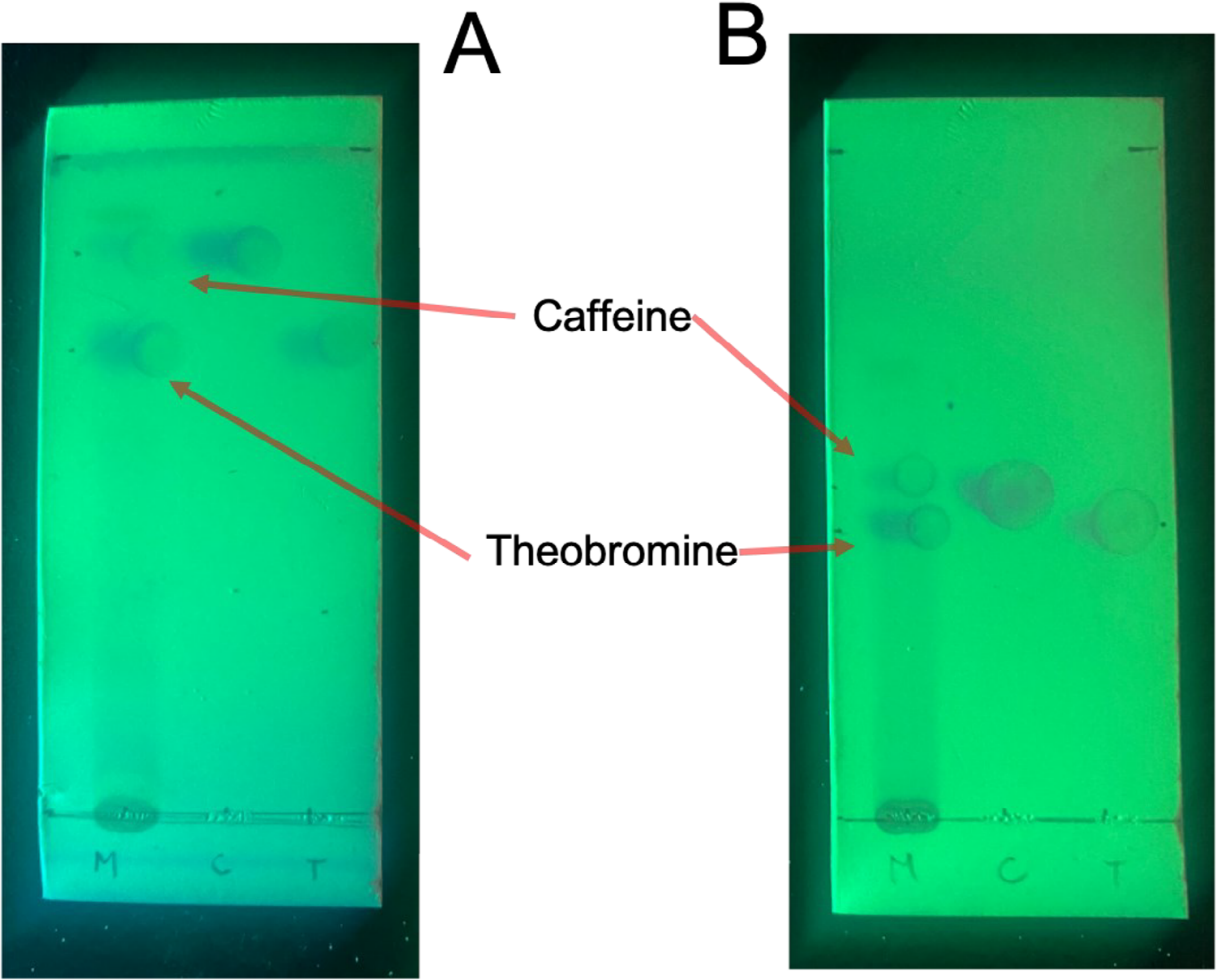
TLC of the samples with two different solvent systems.

The separation of theobromine and caffeine was achieved using TLC. Figure 2 shows the TLC of the eluted sample with Solvent A and with Solvent B, in both three points are observed in the base, one corresponding to the sample and two corresponding to the caffeine and theobromine standards. In the sample eluted with the mixture of Solvent A, better separation is observed compared to the sample eluted with the Solvent B system. The difference in the intensity of the marks when the plate is illuminated with 254 nm light is related to the concentration present in the sample. Table 1 reports the Rf values for each of the spots for both standards and samples. This table shows a correspondence between the Rf of the theobromine and caffeine standards with the marks of those compounds in the sample. For the sample eluted with the solvent mixture A, the difference in the Rf is greater so the separation and determination is greater and more sensitive.

Figure 2 illustrates TLC plates with two different solvent systems. Solvent system A exhibited greater separation between the compounds of interest, so it was the system used in the remaining experiments [1]. While the Rf value provides a valuable reference for determining sample composition, the possibility of co‐eluting compounds with very close Rf values can introduce a degree of uncertainty into the measurements. For this reason, further characterisation using IR and Raman spectroscopy was performed for more precise identification.

### 3.3 FTIR and Raman Experimental and Theoretical Characterisation

**FIGURE 3:**
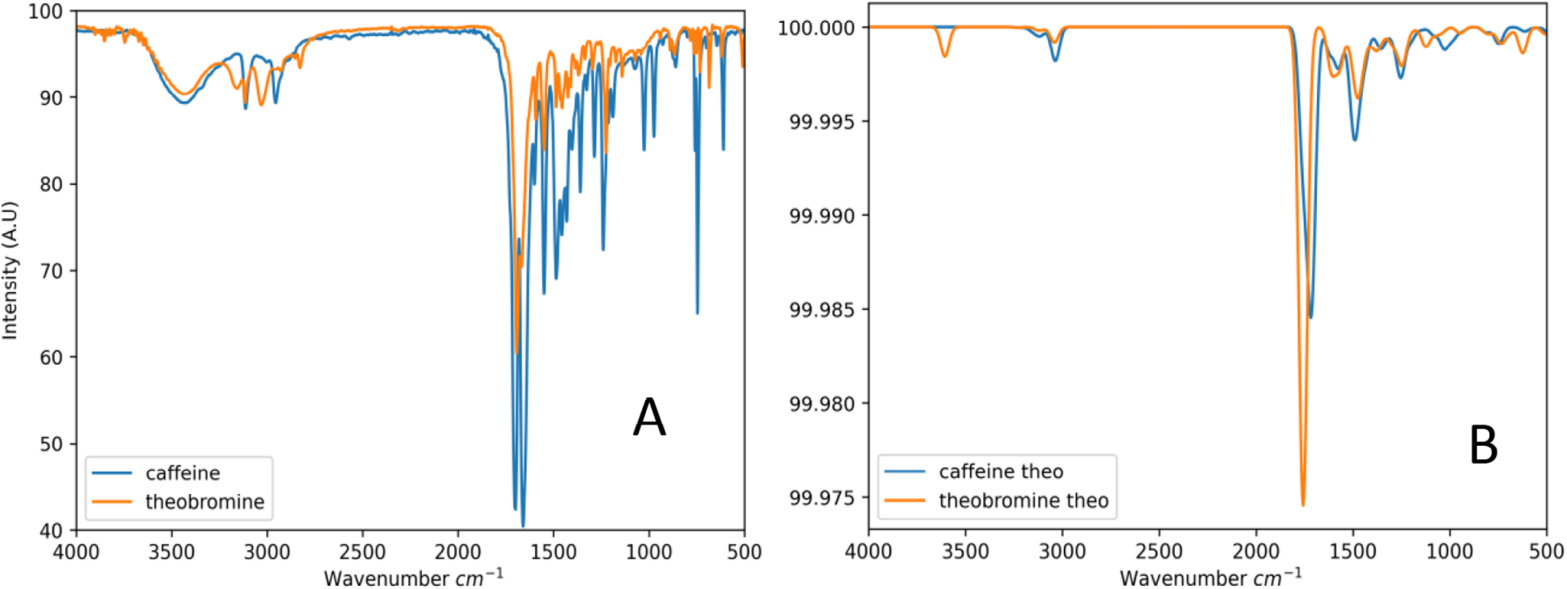
Experimental (A) and theoretical (B) FTIR spectra of theobromine and caffeine.

Figure 3 compares the experimental (A) and theoretical (B) FTIR spectra of theobromine and caffeine. The theoretical spectra were calculated using the methodology described in Section 2.7. Both molecules due their structural similarity exhibit similar vibrational bands. Theobromine differ of caffeine only in the presence of an additional methyl group. The FTIR spectra of the molecules can be divided into four regions: the range from 2000 to 3000 cm^−1^ correspondent to vibrations of C─H and C─N bonds. The range 1700–1500 cm^−1^ for vibrations of C═O, C═N and C═C bonds, the range 1500–1000 cm^−1^ primarily C─N interactions and the range 1000–650 cm^−1^ correspondent to vibrations of N═C─H and N─C─H interactions.

The experimental and theoretical spectra show good agreement, with minor differences in peak intensities due to the absence of solvent effects in the theoretical calculations. The theoretical spectra, calculated in vacuum, tend to overestimate the intensity of N═C─H and N─C─H interactions compared to the experimental spectra obtained in solution.

### 3.4 Theobromine and Caffeine SERS Analysis

The Figure 6 displays the SERS of theobromine and caffeine separated by TLC after dropping AgNPs. The upper spectra is the raw intensity, the lower is the corrected spectra after using Python libraries to baseline correction via asymmetric least squares (ALS) noise reduction with Savitzky–Golay filters. Three spectra are taken and averaged. The spectra show distinct vibrational peaks position and intensity that allow their differentiation. In the low frequency region from 500 to 1000 cm^−1^, theobromine and caffeine spectra shown a weak peak at 641.13 cm^−1^, this peak is associated with skeletal deformation of the aromatic ring or C═C─C deformation, in this zone the theobromine spectra show an additional peak at 732.93 cm^−1^, associated with O═C─C deformation vibrations, these differences have been reported in previous works [19, 25, 26].

In the region from 1000 to 1500 cm^−1^, both compounds show intense peak around 1313 and 1607.75 cm^−1^ corresponding to C─N and CH3 stretching, the relative intensity of these peaks depending on the molecule. Theobromine spectra in the figure show peaks at 732.9 and 1138.61 cm^−1^, which are not present in caffeine spectra and are attributed to methyl group vibrations, whereas caffeine has peaks at 1240.64 and 1538.19 cm^−1^, associate to conjugated C═C or C═N stretching. In the high frequency region from 1500 to 2000 cm^−1^, theobromine and caffeine spectra show peaks at 1607.75 and 1611.59 cm^−1^, respectively, these peaks have been assigned to assigned to v(C═C) + v(C─N) + δ (CH3) stretching, with the slight shift in caffeine due to its additional methyl group. Both spectra also show weak bands in the 2500–3000 cm^−1^ region, corresponding to C─H and N─H stretching, which are less intense possibly due to solvent effects or intermolecular interactions. In addition, the main differences between the spectra, such as the peak positions and intensities of the peaks in the 1200–1400 and 1580–1620 cm^−1^ region, are the more important characteristics in distinguishing theobromine from caffeine. These differences are due to the additional methyl group in caffeine, which modifies the vibrational frequencies and intensities.

**FIGURE 4:**
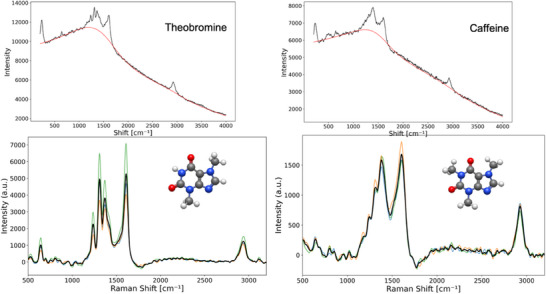
SERS spectra of theobromine and caffeine showing the molecular structures. The upper plot is the uncorrected (raw) spectra and the lower corrected using Python and Matplotlib. The black line correspond to the average spectra of three measurements.

The calculated and experimental Raman spectra for pure caffeine (A) and theobromine (B) are shown in Figure 4. The spectra could be divided into two regions: the short frequency region from 500 to 2000 cm^−1^ (called fingerprint) and the region from 3000 to 4000 cm^−1^ (called high‐frequency). In the fingerprint region are the bands corresponding to vibrations of C─C, C─N and C═O bonds. In the high‐frequency region are present the vibration of the O─H, C─H and N─H bonds. In the figure the difference in the intensity ratio of the peaks at 1611 cm^−1^ (C═C stretching) and 1313 cm^−1^ (C─N stretching) is clearly observed, reflecting their structural differences. These differences are consistent in both theoretical and experimental spectra, validating the computational approach.

The FTIR and Raman SERS spectra of caffeine and theobromine have important similarities although they show differences in some of their characteristic peaks. To determine more precisely the possibility of differentiating these compounds through their spectra, a PCA was performed, for which series of spectra of caffeine and theobromine were taken and a PCA was applied using the Python libraries.

The PCA of SERS for caffeine (Group C) and theobromine (Group T) is showed in the Figure 7. This plot reveals distinct clustering along the axis of PC1 (Principal Component 1), which range from −60 to 40, indicating that this component captures the primary structural differences between the two alkaloids spectra. The plot show a clear separation of the groups along PC1 which reflects the main molecular spectra distinctions. The secondary component, PC2 (−40 to 40), accounts for smaller variations, possibly linked to experimental noise or minor sample heterogeneity. This PCA demonstrates the utility of SERS Raman spectroscopy combined with multivariate analysis for rapid, non‐destructive discrimination of structurally similar alkaloids.

**FIGURE 5:**
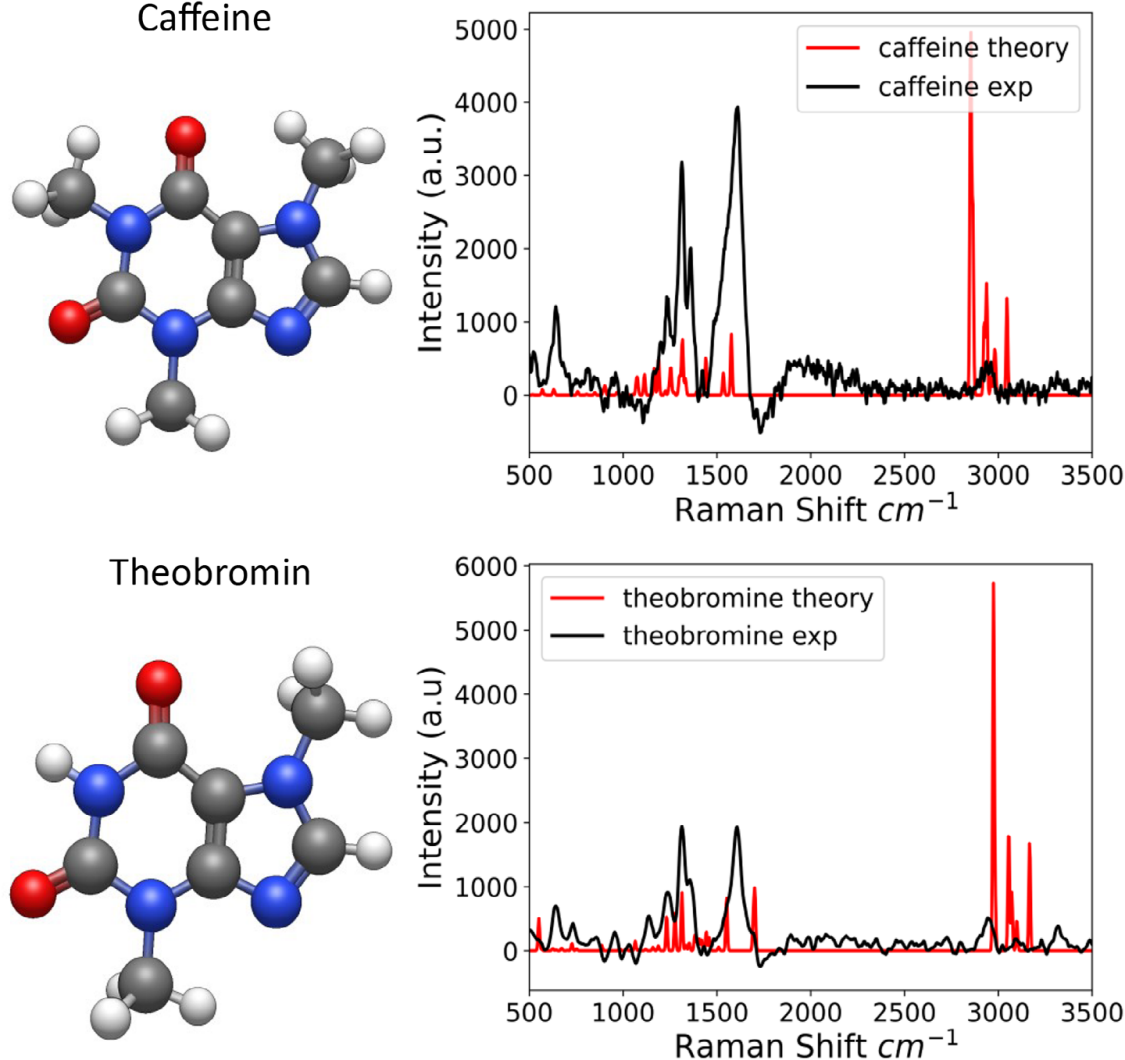
Theoretical and experimental Raman spectra for caffeine and theobromine.

**FIGURE 6:**
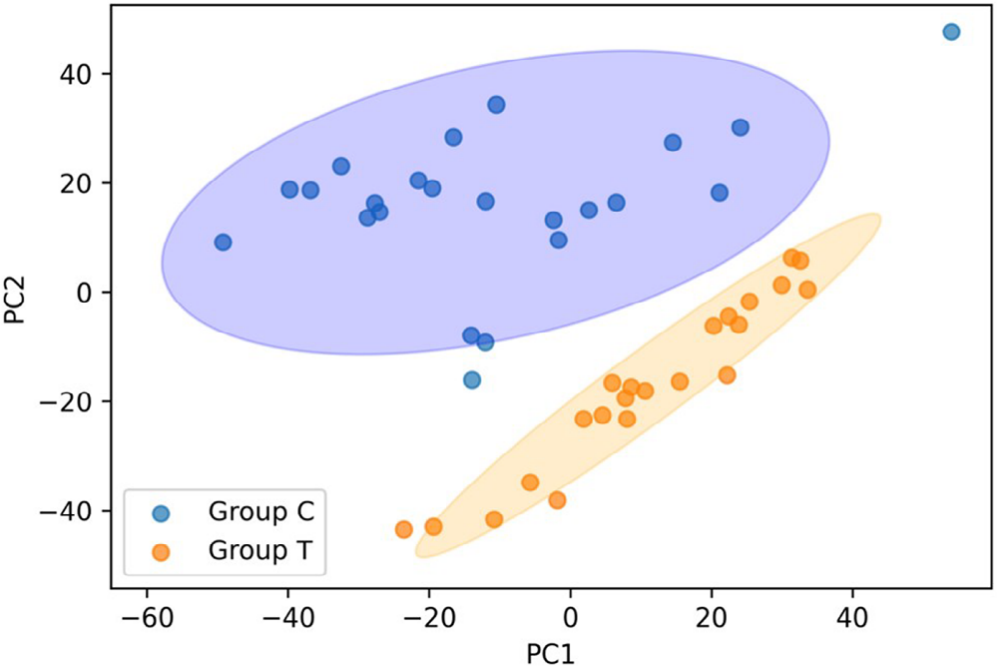
Principal component analysis (PCA) of SERS for caffeine (Group C) and theobromine (Group T).

**FIGURE 7:**
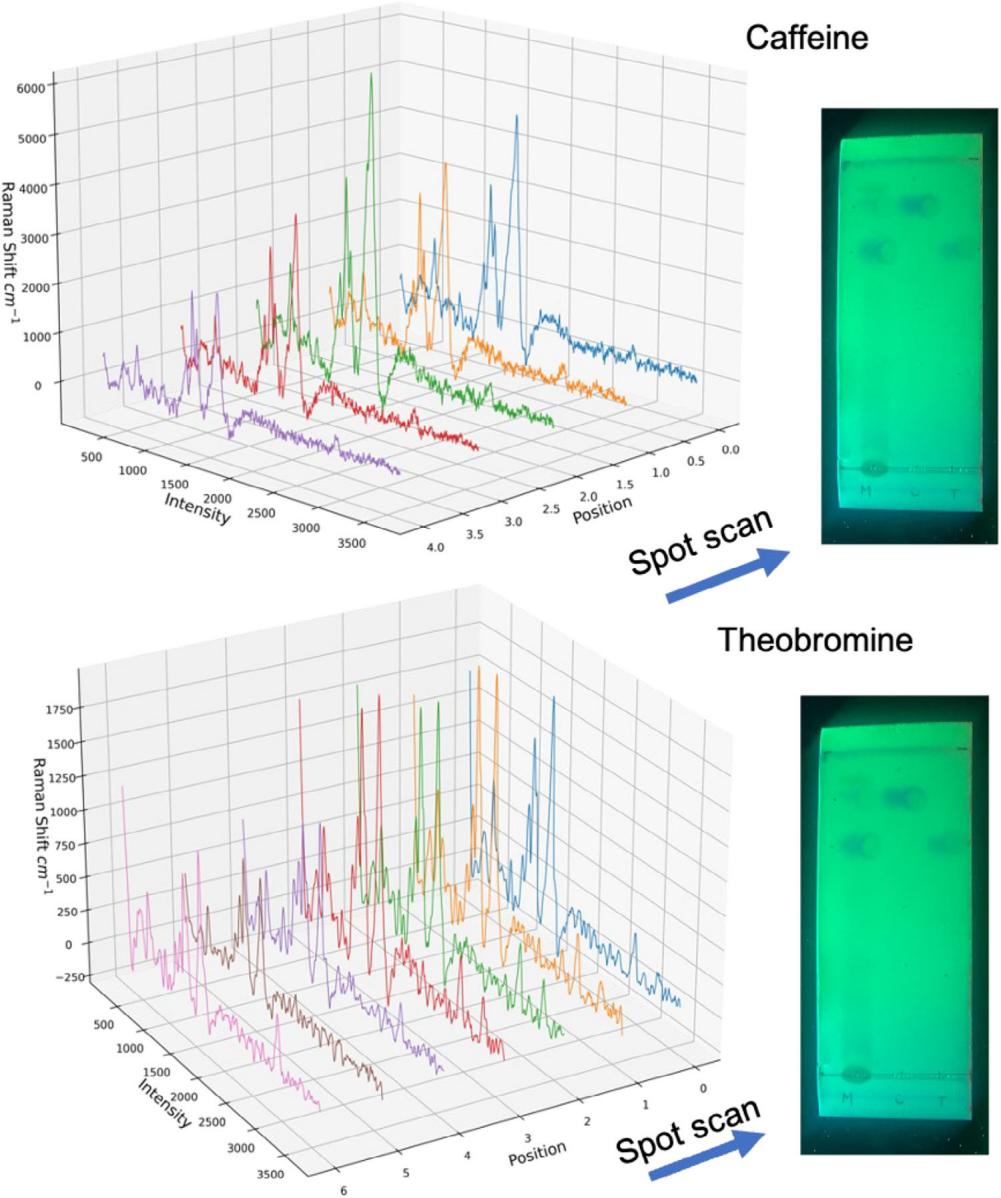
TLC separation with SERS detection for cocoa pods extract sample SERS scanning of the spot for caffeine and theobromine.

The main spectroscopic basis for the separation and classification observed in the PCA analysis of Figure 6 lies in the distinctive vibrational peaks and their intensity differences in the SERS spectra of theobromine and caffeine. Despite the great structural similarity between theobromine and caffeine, which differ only by an additional methyl group, this subtle structural difference translates into significant variations in their vibrational frequencies and intensities, which are effectively captured by Raman spectroscopy. Key differences observed in the SERS spectra (Figure 5) that serve as the basis for the separation in PCA include the presence of an additional peak at 732.93 cm^−1^ in theobromine (associated with O═C─C strain vibrations) that is absent in caffeine. Furthermore, the relative intensities of peaks such as 1313 cm^−1^ (C─N stretch) and 1607.75 cm^−1^ (CH_3_ stretch) differ between the two molecules. Theobromine also exhibits specific peaks at 732.9 and 1138.61 cm^−1^ (attributed to methyl group vibrations) that are not found in caffeine, while caffeine exhibits peaks at 1240.64 and 1538.19 cm^−1^ (associated with C═C or C═N conjugate stretches). Finally, a slight shift in the caffeine peak (1611.59 cm^−1^) is observed compared to theobromine (1607.75 cm^−1^) in the high frequency region, which is attributed to the presence of its additional methyl group. By reducing the multidimensional spectral data set, PCA enables PC1 to capture these primary structural differences, which is manifested in the clear separation of the groups in Figure 7, even when the raw spectra may appear similar to the naked eye.

PCA is a powerful tool for dimensionality reduction and pattern visualisation in complex data sets. Its advantages include its unsupervised nature, making it ideal for exploratory data differentiation without the need for predefined class information. Furthermore, it is very effective at capturing maximum variance in the data, such as the subtle differences caused by methyl group vibrations in caffeine and theobromine. However, the PCA also presents limitations; for example, the interpretability of its payloads can be complex, and is sensitive to data scaling and preprocessing. Being an unsupervised method, it does not use information from class labels to maximise separation between groups, which can be a disadvantage for specific classification tasks.

Exist alternatives PCA for multivariate analysis in spectroscopy. Partial least squares discriminant analysis (PLS‐DA) is a supervised method best suited for classification tasks and for identifying discriminative biomarkers, as it maximises the separation between sample classes. However, PLS‐DA requires labelled training data and is more prone to overfitting, especially with small or noisy data sets. Other techniques include t‐SNE (t‐distributed stochastic neighbour embedding), which is better for visualising nonlinear separations and complex structures in high‐dimensional data, although it is computationally intensive and less interpretable in terms of feature importance. In this study, PCA was the appropriate choice for validating the distinction of compounds, given its effectiveness in the initial exploratory differentiation and the nature of the observed spectral differences.

### 3.5 TLC‐SERS Analysis of Cocoa Extract Sample

The combination of TLC separation with SERS detection was applied to analyse a cocoa bean extract. Figure 7 shows the TLC plates where a 2 µL drop of the extract was seeded and run according to the methodology described above, the image shows a clear differentiation between the spots of caffeine and theobromine. SERS analysis was performed by depositing 2 µL of AgNPs solution onto the TLC spots and scanning with the laser beam and recording the spectra at different points. The intensity of the SERS signal varied across the spots, likely due to the inhomogeneous distribution of the compounds during the separation. For both cases the spectra increase in intensity until they reach a maximum and then reach a minimum, maintaining the structure. Spectra clearly show their differences for theobromine and caffeine, demonstrating the potential of TLC‐SERS for the analysis of complex samples. The results confirm the need to carefully scanning each separate spot, select and average the spectra to have a reproducible signal. To quantitatively validate spectral reproducibility across multiple scans, triplicate measurements were performed and the relative standard deviations (RSDs) of key peak intensities and their positions were evaluated, confirming the high reproducibility of the methodology.

## 4 Preliminary Quantitative Analysis

**FIGURE 8:**
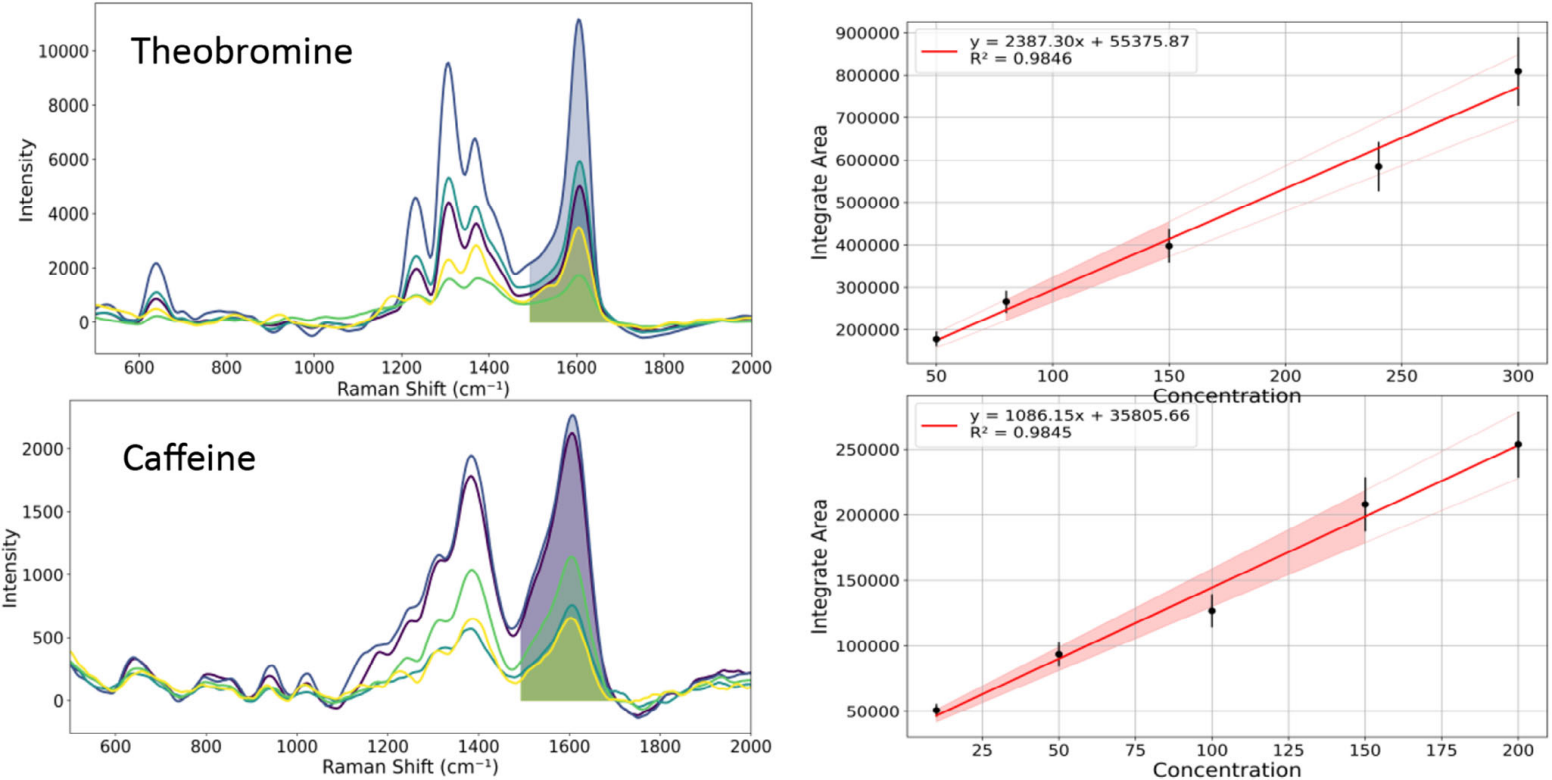
Integrate area of SERS spectra for caffeine and theobromine, and calibration curves calculated by plotting integrate area versus concentration.

Although the primary objective of this study was to establish a robust methodology for the unequivocal identification and differentiation of theobromine and caffeine in cocoa extracts, a section with preliminary quantitative data has been included. This inclusion is intended to demonstrate the potential of the TLC‐SERS methodology for future applications in the quantification of these alkaloids in cocoa. Quantification is recognised as an important aspect of analysis, and this section serves as a proof of concept, laying the groundwork for more detailed and comprehensive work focused on the development and validation of accurate quantitative methods. Figure 8 shows calibration curves for theobromine and caffeine. Excellent linearity was observed (*R* ^2^ = 0.9845 for both), validating TLC‐SERS for quantification. The slopes reflect higher SERS sensitivity for theobromine (2387.30 vs. 1086.15 intensity·L/mg), attributed to stronger AgNP interactions with its N─H group (Figure 5). LODs were calculated as 3.3σ/S, yielding (0.5 mg/L) for theobromine and (1.1 mg/L) for caffeine. When applied to cocoa extracts, concentrations were 83 mg/g (theobromine) and 64 mg/g (caffeine), in good agreement with the literature.

## 5 Conclusions

The applicability of integration of TLC for separation and SERS using AgNPs for identification of theobromine and caffeine in cocoa samples has been demonstrate. The synthesised AgNPs (∼20 nm) exhibited optimal SERS activity, enabling the detection of the different molecules in TLC plates. TLC efficiently separated the compounds, with distinct Rfs facilitating their preliminary identification in complex matrices. DFT calculations at the r2SCAN‐3c level provided a robust theoretical framework, allowing the comparison with experimental FTIR and Raman spectra. The differences between theoretical and experimental data obtained were attributed to solvent effects and intermolecular interactions. The PCA was employed to statistically differentiate the spectral profiles of caffeine and theobromine. Reducing the multidimensional spectral data into principal components, PCA allow to discriminate and unambiguously distinguishing the two compounds. This multivariate approach confirmed the reliability of SERS and FTIR data, addressing a key challenge in analysing structurally analogous molecules. The TLC‐SERS platform, combined with PCA, offers a cost‐effective, sensitive and comprehensive strategy for analysing samples of cocoa and derivates. By reducing the multidimensional spectral data into principal components, PCA allowed the two compounds to be unambiguously discriminated and distinguished, based on their distinctive vibrational fingerprints, such as the additional peak at 732.93 cm^−1^ in theobromine and the differences in the relative intensities of key peaks in the 1200–1400 and 1580–1620 cm^−1^ regions. This multivariate approach confirmed the reliability of the SERS and FTIR data, addressing a key challenge in the analysis of structurally analogous molecules. The TLC‐SERS platform, combined with PCA, offers a cost‐effective, sensitive and comprehensive strategy for the analysis of cocoa and cocoa derivative samples. The methodology presented in this work opens the possibility of rapid and accurate identification of theobromine and caffeine in cocoa products, which is of great value for quality control and origin assessment, especially in resource‐limited settings. Furthermore, this methodology lays a solid foundation for its potential applicability to other food matrices where rapid and cost‐effective identification and differentiation of structurally similar compounds is crucial. The method enables quantification of theobromine (LOD = 0.5 mg/L) and caffeine (LOD = 1.1 mg/L) via calibration curves (*R* ^2^ > 0.98), providing a cost‐effective alternative to HPLC for cocoa quality control. Future research is anticipated to focus on the precise quantification of these alkaloids, taking advantage of the robustness of this platform.

## Author Contributions

**Jimmy Castillo**: conceptualization, funding acquisition, writing review & editing. **Maria Rodriguez**: conceptialization, methodology, resources. **Romel Guzman**: resources, supervision. **Briggit Katan**: investigation (equal). **Ray ARteaga**: investigation (equal). **Maria Figueira**: investigation (equal).

## Conflicts of Interest

The authors declare no conflicts of interest.

## Supporting information




**Supporting file 1**: ansa70033‐sup‐0001‐SuppMat.docx

## Data Availability

All data generated or analysed during this study are included in this published article and the Supporting Information. Raw data (SERS spectra, computational outputs) are available upon reasonable request to the corresponding author.
