# Supplementary material for: Separation and Determination of Theobromine and Caffeine in Cocoa Beans Extract Using TLC‐SERS: Identification and Computational Insights
Source: Anal Sci Adv. 2025 Jul 28;6(2):e70033. doi: 10.1002/ansa.70033 (PMC12303255; doi:10.1002/ansa.70033)

Supplemental

1. Additional Raman intensity plots for caffeine and theobromine, original and after adjustment.


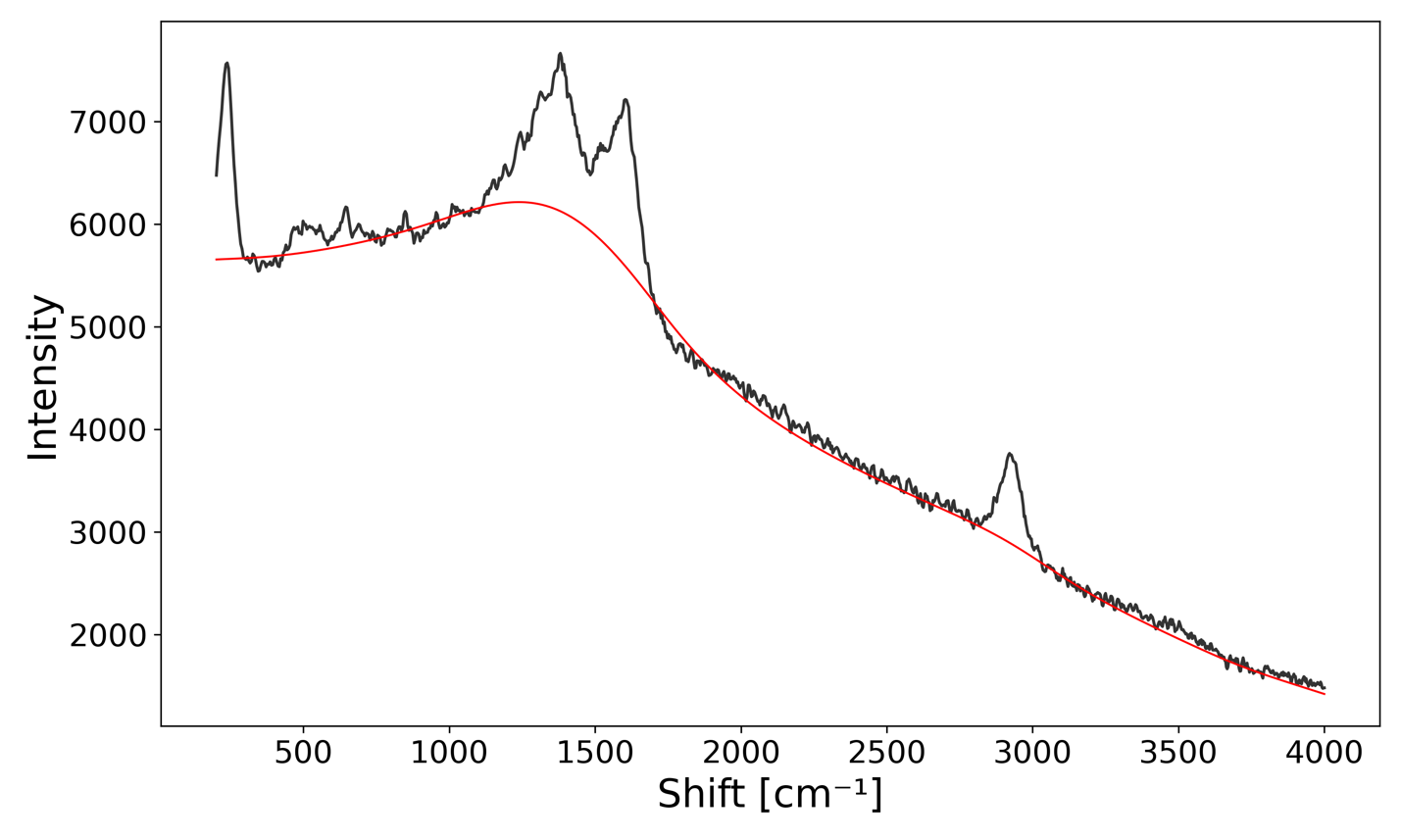

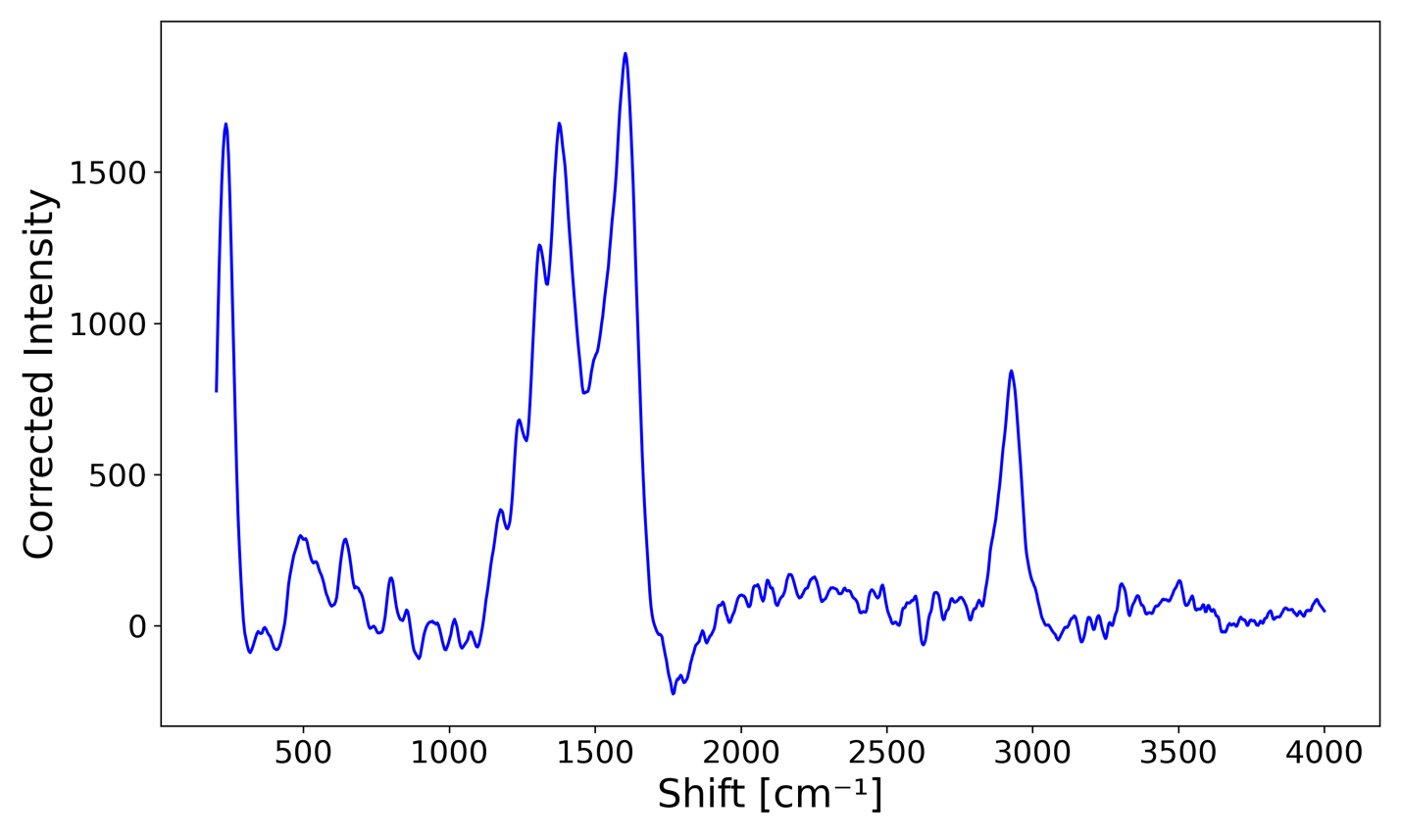


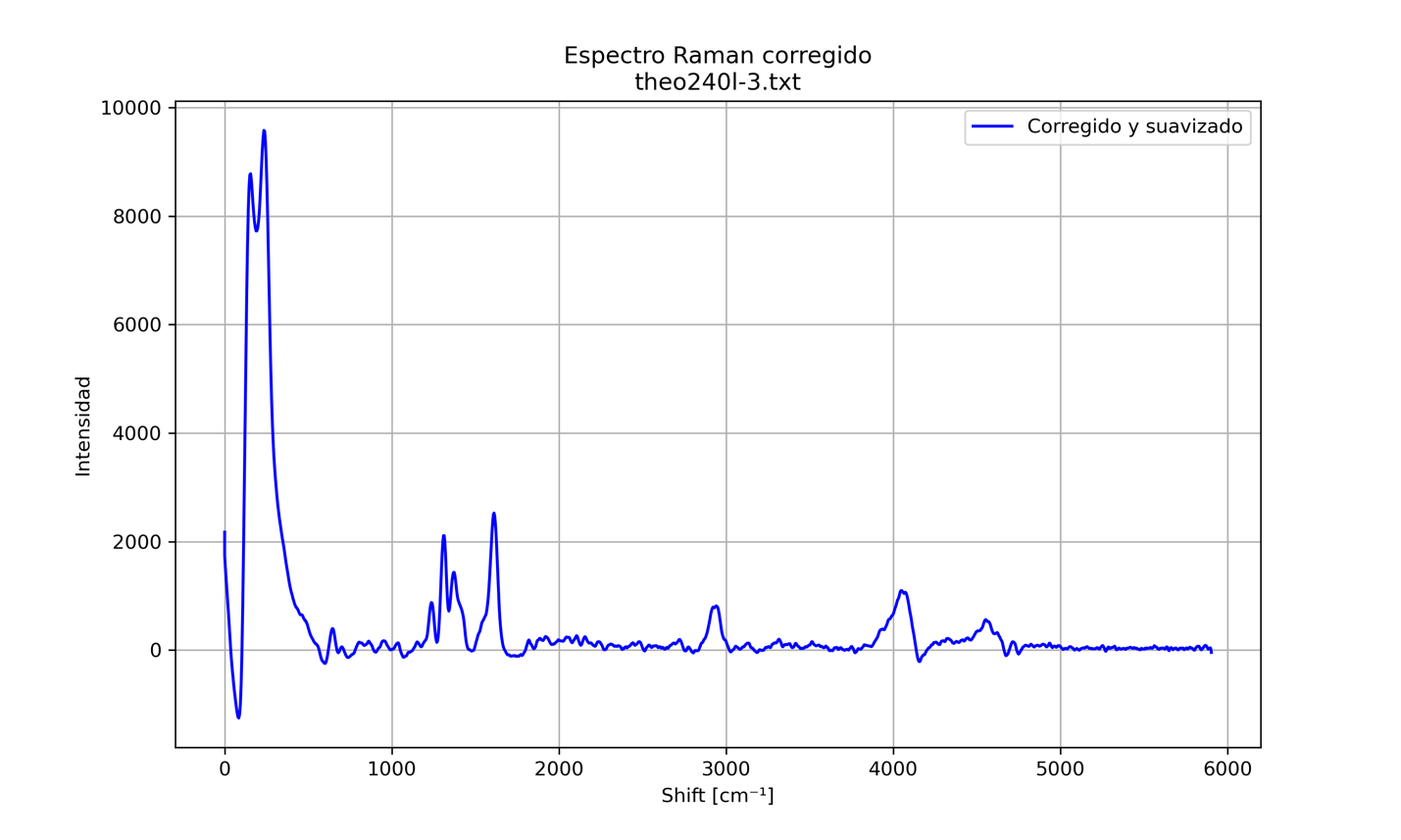

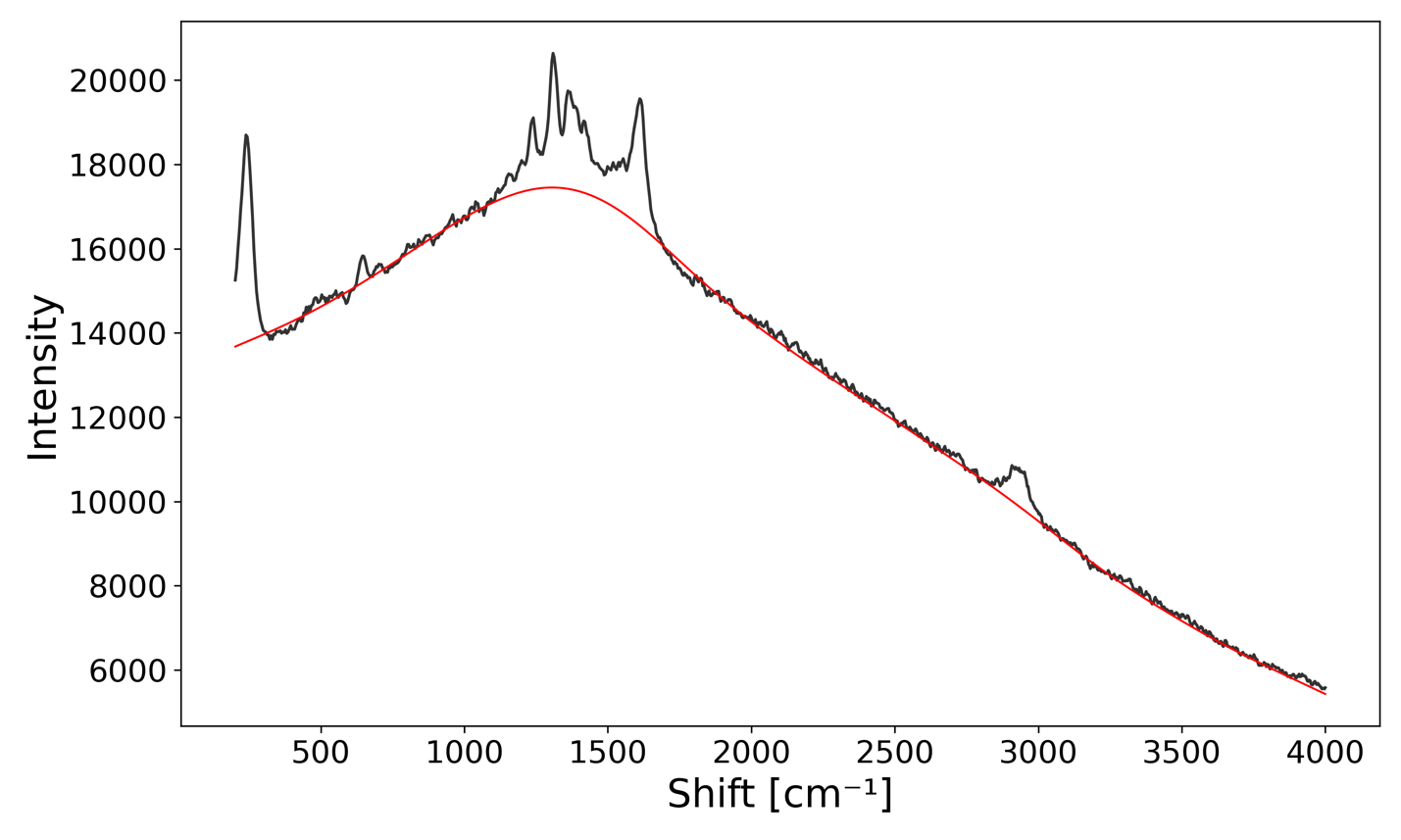


1. Calculation of HOMO and LUMO orbitals for caffeine and theobromine molecules. Calculation of the energy difference between the orbitals, which shows the origin of the difference in the intensities of the spectrum and the points available to interact with the nanoparticles.


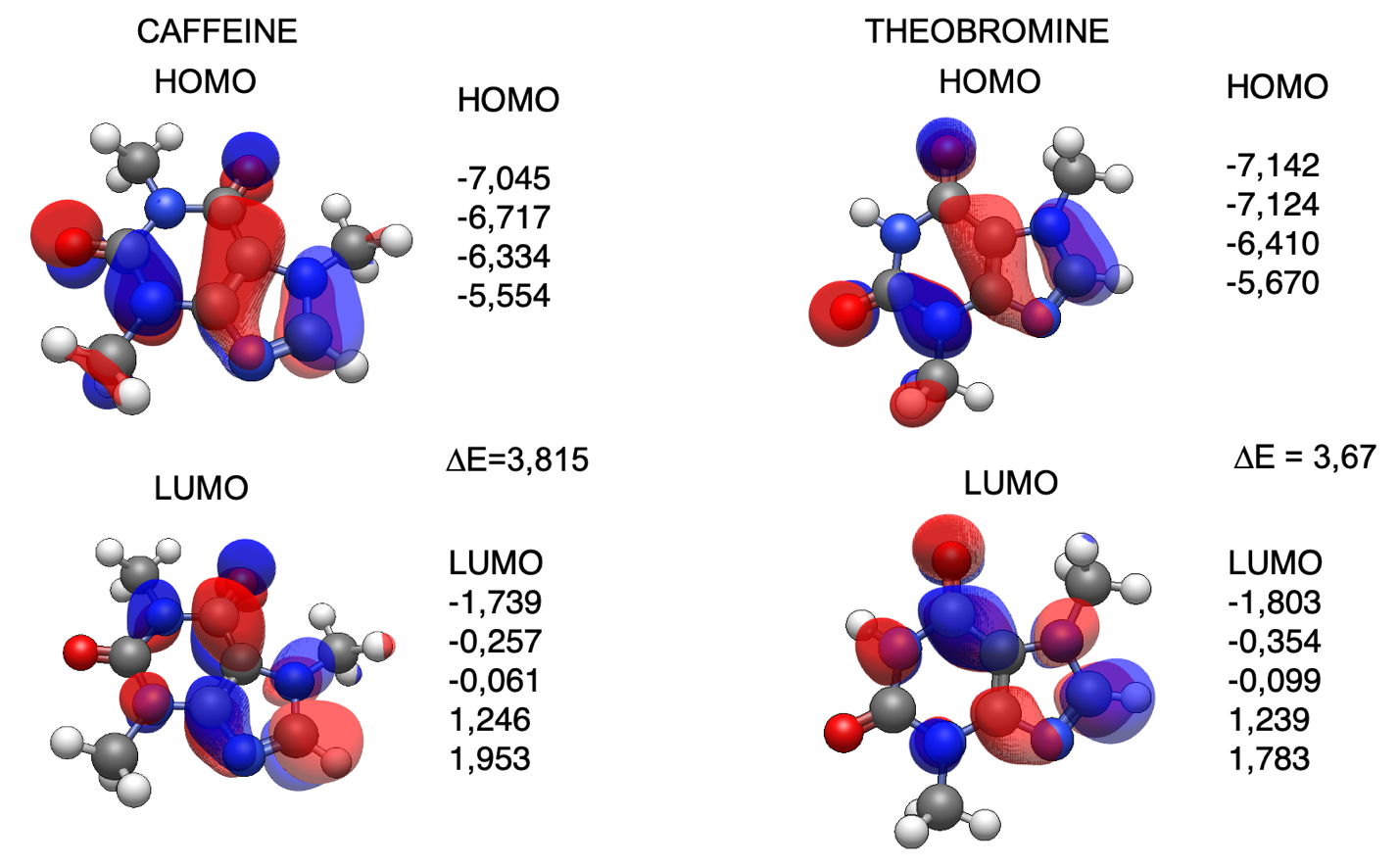


1. Loadings calculations from PAC data showing the zones of interest.


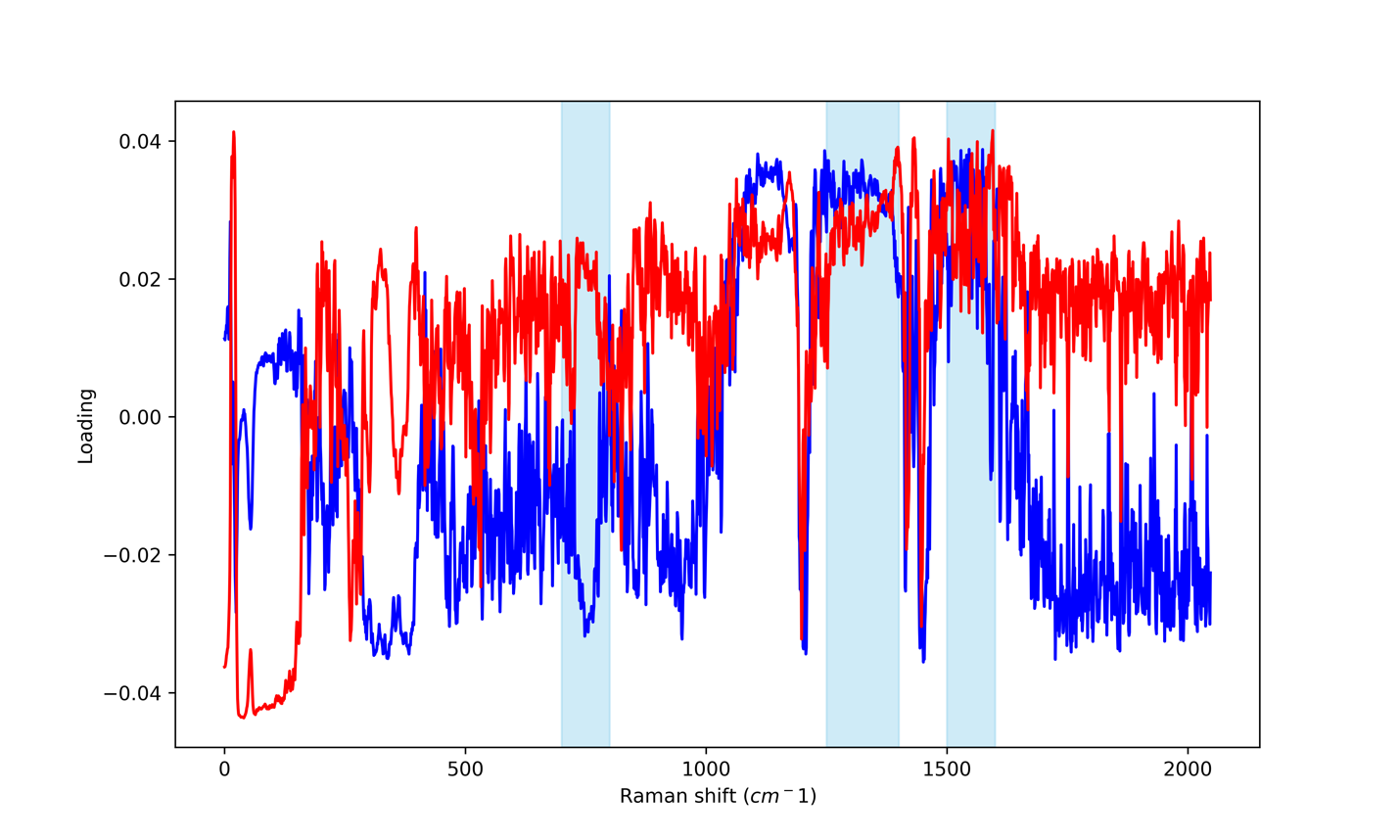


1. Integrated areas for another set of caffeine and theobromine solutions


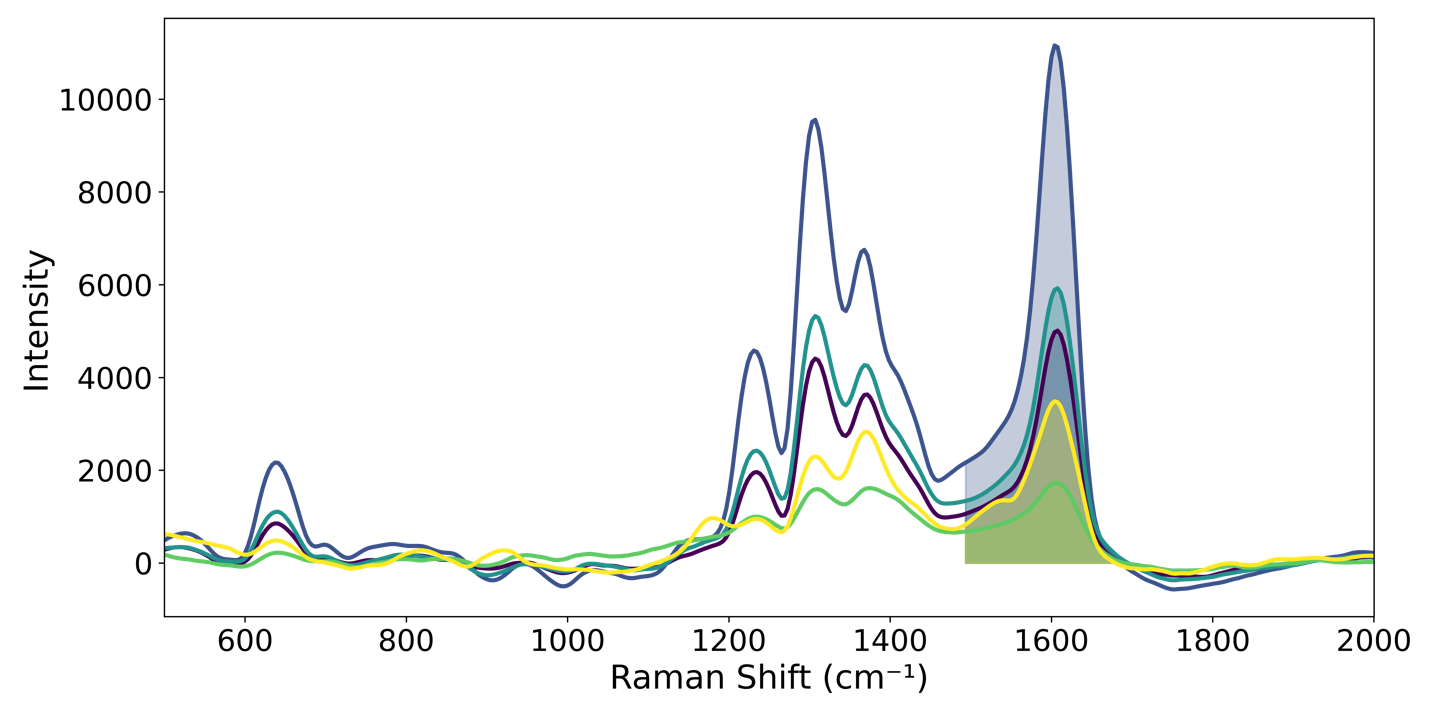


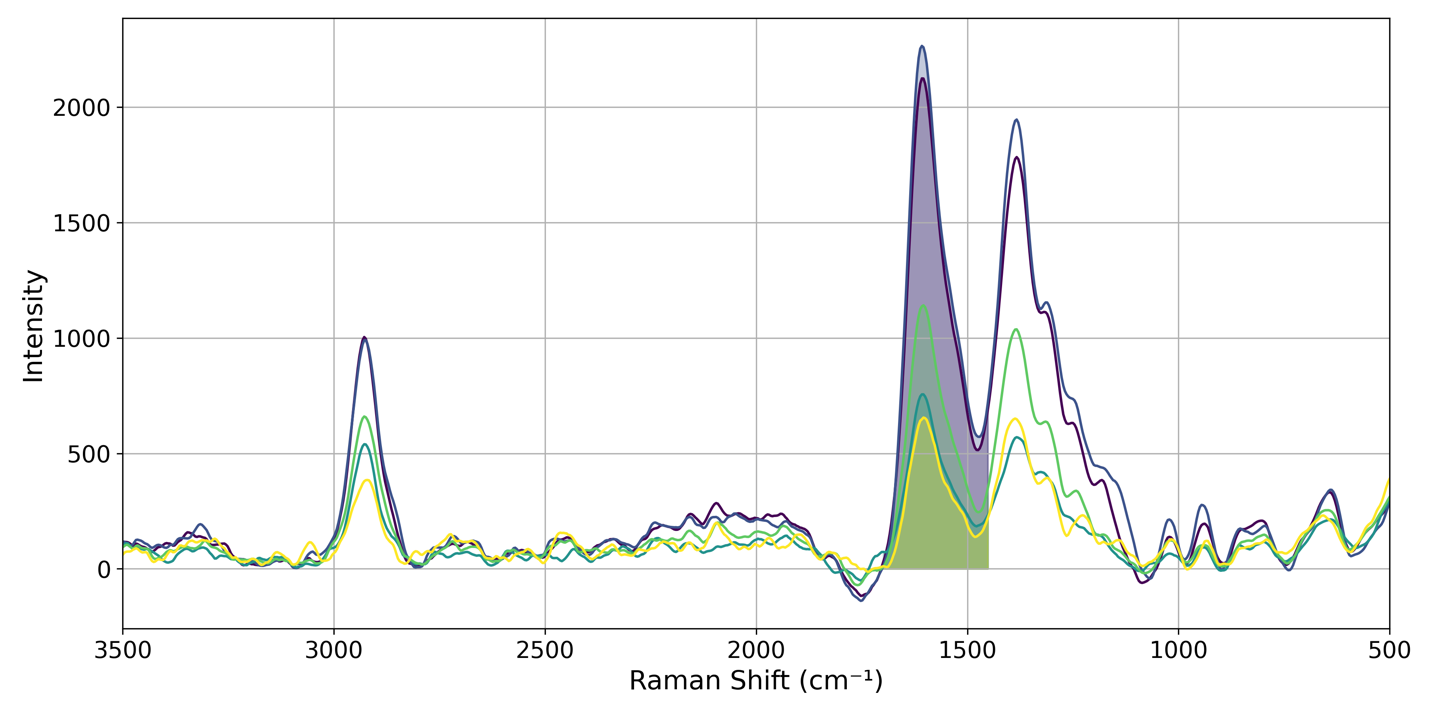


1. Spectra for caffeine and theobromine samples, their triplicates and the average.


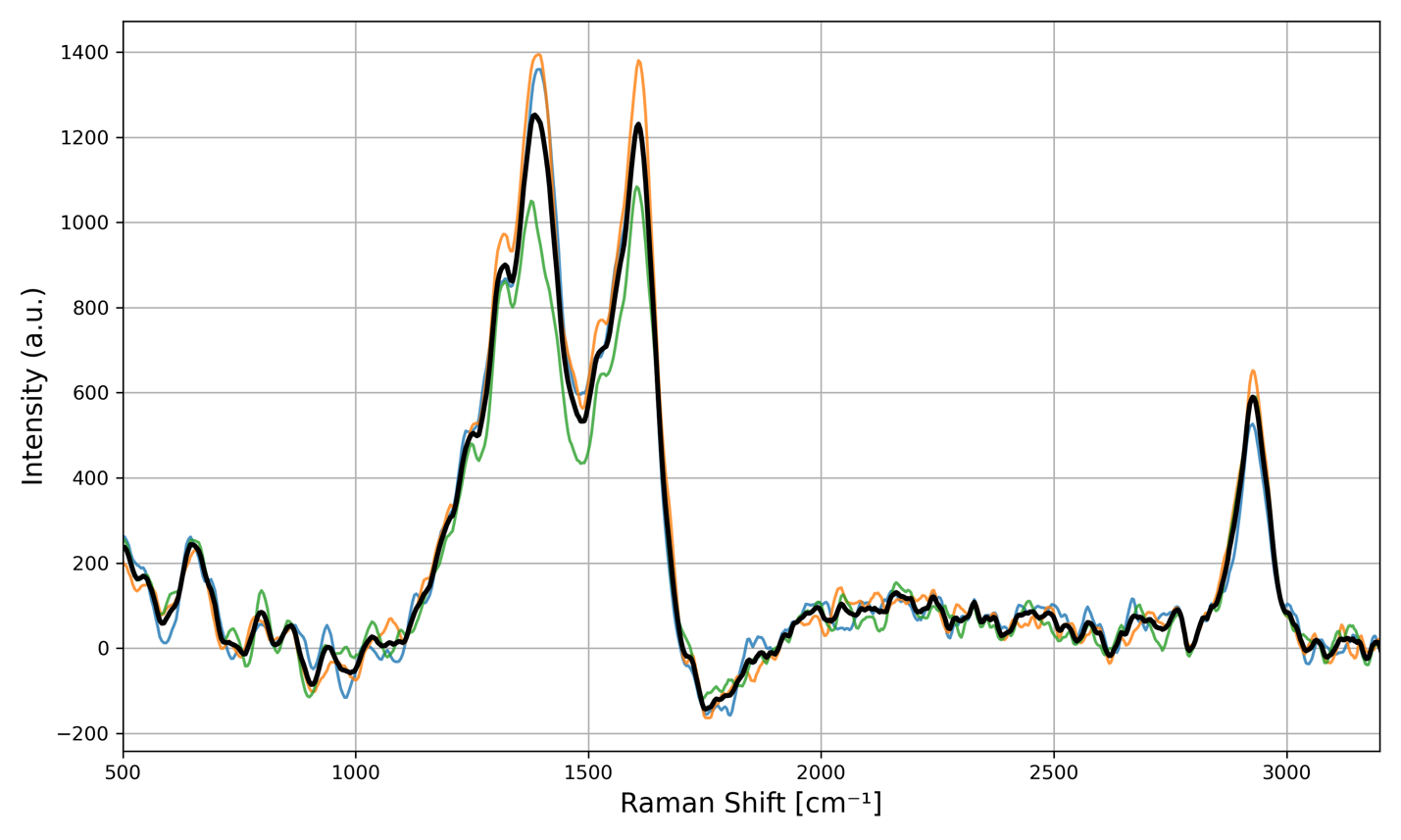


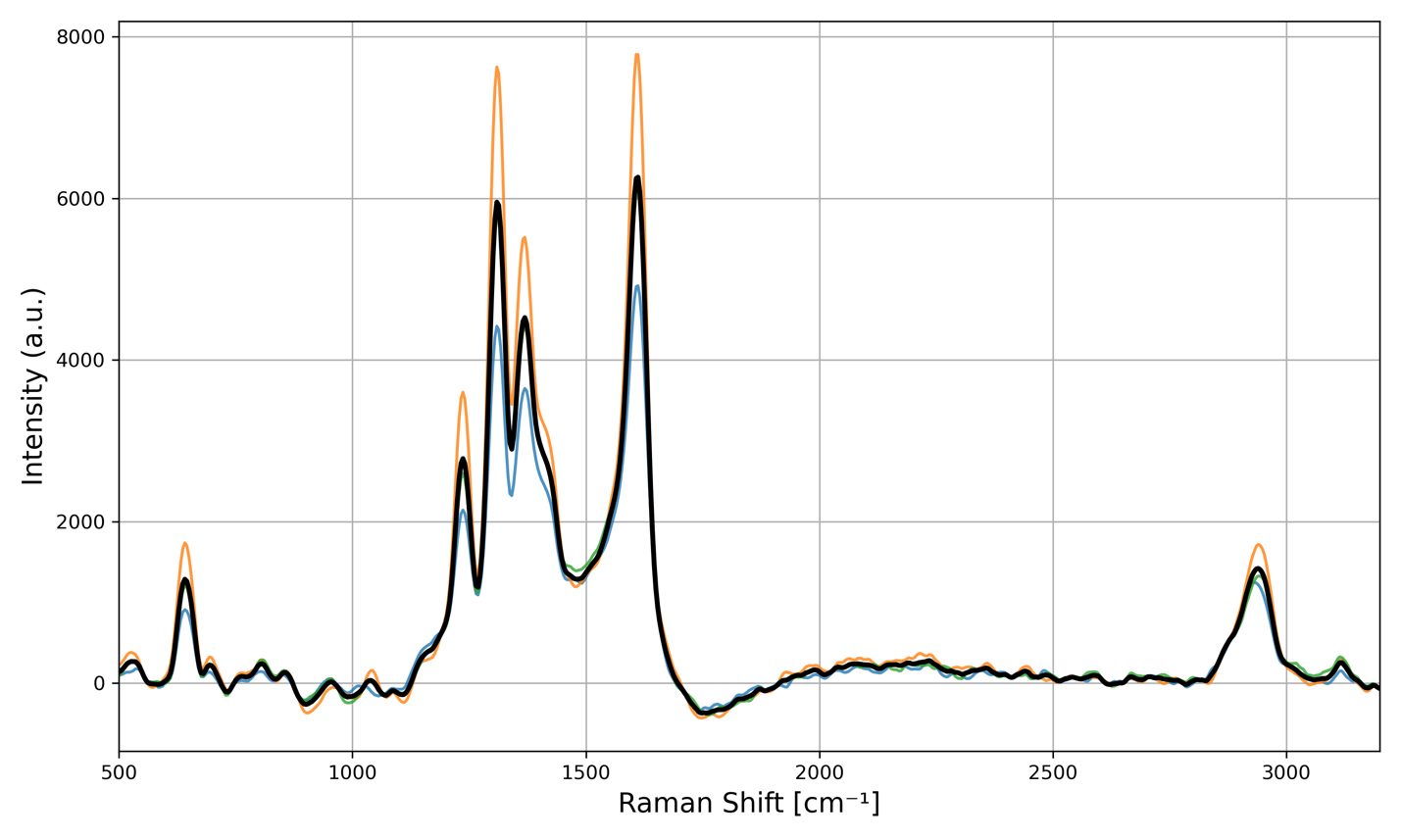

Supplement: Supplementary file 1 — Supporting file 1: ansa70033‐sup‐0001‐SuppMat.docx [file ANSA-6-e70033-s001.docx]
